# Metagenomic Functional Profiling Reveals Differences in Bacterial Composition and Function During Bioaugmentation of Aged Petroleum-Contaminated Soil

**DOI:** 10.3389/fmicb.2020.02106

**Published:** 2020-08-31

**Authors:** Magdalena Pacwa-Płociniczak, Paulina Biniecka, Kinga Bondarczuk, Zofia Piotrowska-Seget

**Affiliations:** ^1^Institute of Biology, Biotechnology and Environmental Protection, Faculty of Natural Sciences, University of Silesia in Katowice, Katowice, Poland; ^2^Centre for Bioinformatics and Data Analysis, Medical University of Bialystok, Białystok, Poland

**Keywords:** petroleum hydrocarbons, bioaugmentation, soil microbial communities, high-throughput sequencing, metagenome prediction

## Abstract

Our objective was to study the bacterial community changes that determine enhanced removal of petroleum hydrocarbons from soils subjected to bioaugmentation with the hydrocarbon-degrading strains *Rhodococcus erythropolis* CD 130, CD 167, and their combination. To achieve this, a high-throughput sequencing of the 16S rRNA gene was performed. The changes in the bacterial community composition were most apparent the day after bacterial inoculation. These changes represented an increase in the percentage abundance of *Rhodococcus* and *Pseudomonas* genera. Surprisingly, members of the *Rhodococcus* genus were not present after day 91. At the end of the experiment, the bacterial communities from the CD 130, CD 167, and control soils had a similar structure. Nevertheless, the composition of the bacteria in the CD 130 + CD 167 soil was still distinct from the control. Metagenomic predictions from the 16S rRNA gene sequences showed that the introduction of bacteria had a significant influence on the predicted pathways (metabolism of xenobiotics, lipids, terpenoids, polyketides, and amino acids) on day one. On day 182, differences in the abundance of functional pathways were also detected in the CD 130 and CD 130 + CD 167 soils. Additionally, we observed that on day one, in all bioaugmented soils, the *alkH* gene was mainly contributed by the *Rhodococcus* and *Mycobacterium* genera, whereas in non-treated soil, this gene was contributed only by the *Mycobacterium* genus. Interestingly, from day 91, the *Mycobacterium* genus was the main contributor for the tested genes in all studied soils. Our results showed that hydrocarbon depletion from the analyzed soils resulted from the activity of the autochthonous bacteria. However, these changes in the composition and function of the indigenous bacterial community occurred under the influence of the introduced bacteria.

## Introduction

Total petroleum hydrocarbons (TPH) are a broad family of organic compounds that originate from crude oil and contain many carcinogens and neurotoxic pollutants (Ławniczak et al., 2020). According to the European Environment Agency (2017), the pollution caused by petroleum products is considered to be one of the most serious global environmental problems.

Since exploiting bacteria for the removal of organic pollutants from the environment has shown great promise, the development of an effective bioremediation strategy for soils contaminated with hydrocarbons is one of the major tasks facing environmental microbiologists. Bioaugmentation is a technique in which selected microbial strains are introduced into a contaminated environment to increase the rate of pollutant removal (Vogel, 1996). It is believed that the efficiency of this method strongly depends on the ability of the inoculants, which are equipped with the appropriate degradation pathways, to remain active in the contaminated environment (Festa et al., 2016; Płociniczak et al., 2017). The poor survivability and adaptability of inoculants often results in the failure of a bioaugmentation strategy (Szulc et al., 2014). Thus, to extend the survival of the inoculants, the use of indigenous strains well adapted to the remediated soil, is highly recommended.

However, the application of autochthonous (native) bacterial degraders is unfortunately not always effective, probably because the inoculants often do not exhibit the same activity in the soil as under laboratory conditions (Boon et al., 2002; Pacwa-Płociniczak et al., 2016a). Moreover, the unexplored interactions between introduced bacteria and the autochthonous bacterial communities in the remediated soil could be critical for the success of bioaugmentation (El Fantroussi and Agathos, 2005).

Soil bacteria are known to be social organisms that live in complex communities with extensive interactions within and between species (Stubbendieck et al., 2016). Bioaugmentation can alter these interactions resulting in either a positive or a negative impact on the bioremediation efficiency (Herrero and Stuckey, 2015). Some studies showed that changes in the structure of the bacterial communities in soil polluted with hydrocarbons can promote the efficient degradation of pollutants (Liu et al., 2018; Siles and Margesin, 2018). Introduction of hydrocarbon-degrading and biosurfactant-producing bacteria can stimulate hydrocarbon degradation by the indigenous bacteria by increasing the availability of intermediates that can be used as sources of carbon and energy by autochthonous bacteria and by increasing the bioavailability of hydrophobic compounds (Fathepure et al., 2014; Santos et al., 2016). On the contrary, unfavorable changes can affect the number and activity of hydrocarbon-degrading bacteria, and thus, reduce the rate of pollutant removal. Therefore, the above scenarios should be considered when designing an effective strategy for the bioaugmentation of hydrocarbon-polluted environments. Monitoring the changes in the metabolic activity and structure of the soil bacterial communities during the bioremediation process is essential. While various methods have already been used to characterize bacterial communities, they usually provide information about a chosen group of microorganisms or their selected activities/features. The advent of high-throughput sequencing technology has revolutionized the study of environmental microbiology (Logares et al., 2012). Next-generation sequencing (NGS) methods allow us to study the entire community of microorganisms inhabiting an environment. They provide an opportunity to explore unculturable microorganisms, and more importantly, they allow the study of biological responses to the bioaugmentation process (Roy et al., 2018; Woźniak-Karczewska et al., 2019). However, most studies on ecological processes, especially investigations on bioremediation, concentrate on the analysis of microbial community composition based on the 16S rRNA gene, omitting the functional and metabolic properties of the same (Mukherjee et al., 2017). Recently, the implementation of advanced bioinformatic approaches such as the Phylogenetic Investigation of Communities by Reconstruction of Unobserved States (PICRUSt), a computational approach to predict the functional composition of a metagenome using 16S rRNA gene data and a database of reference genomes, allow us to study the functional capacity of microbial communities with reasonable precision and confidence (Langille et al., 2013). The PICRUSt tool was used to study the differences in bacterial function due to long-term agricultural practices, to assess key bacterial traits for rhizosphere colonization, as well as to demonstrate the functional development of a heavy metal-exposed soil microbial community (Lopes et al., 2016; Dube et al., 2019; Fajardo et al., 2019). In our study, we used the PICRUSt approach to describe the functional composition of bacterial communities inhabiting long-term hydrocarbon contaminated soil subjected to bioaugmentation.

In our earlier study, we analyzed the influence of bioaugmentation on petroleum-polluted soil with the bacterial strains *Rhodococcus erythropolis* CD 130, CD 167, and their combination. We studied their effect on hydrocarbon degradation in the soil, as well as expression levels of selected bacterial genes and the bacterial community structure in the soil over 182 days (Pacwa-Płociniczak et al., 2019). The obtained results did not explain the bacterial responses in the soil, and more detailed analysis was needed. Therefore, this study aimed to complement our previous work by studying the taxonomic composition of the bacterial communities in soils subjected to bioaugmentation with the single hydrocarbon-degrading bacterial strains *R. erythropolis* CD 130, CD 167, and their combination. Additionally, we performed a predictive analysis of the metagenome of the bacterial communities based on their 16S rRNA sequences and determined the contributions of the various taxa to KEGG orthologs (KOs) known to be involved in hydrocarbon degradation.

## Materials and Methods

### Sample Collection

The soil samples were collected from aged petroleum-contaminated soil subjected to bioaugmentation with the hydrocarbon-degrading bacterial strains *Rhodococcus erythropolis* CD 130, CD 167, and their combination. Aged petroleum-contaminated soil was obtained from an industrial area located around a 100 year old refinery in Czechowice-Dziedzice, Upper Silesia, Southern Poland. Over a hundred years of uninterrupted use of crude oil refinery technology based on sulfuric acid has generated tons of acidic petroleum sludge that have been deposited in the waste lagoon. The soil used for bioaugmentation experiment was collected from the area adjacent to the lagoon (top layer, 0–20 cm depth). Soil was classified as silty clay loam (sand 31 ± 3.1%; silt 45 ± 4.5%; caly 24 ± 2.4%) with the following properties: density 1.145 ± 0.002 g cm^–3^; pH_H2O_ 4.02 ± 0.01; organic matter 6.81 ± 0.03%; N_tot_ 0.079 ± 0.001%; C_org_ 1.58 ± 0.12%; P 505.40 ± 29.32 mg kg^–1^; Fe 20740.00 ± 782.56 mg kg^–1^. Before starting the experiment, the soil was air-dried and passed through a 1.2 mm sieve. The pot experiment was conducted at room temperature (22°C) under laboratory conditions using 300 g of contaminated soil per container (17 cm high, 13 cm diameter). The soil moisture was adjusted to 50% of the maximum water holding capacity and maintained at this level during the entire experimental period.

The initial TPH concentration in the soil was 11980.75 ± 833.11 mg kg^–1^ dry weight (d.w.) of soil. The bioaugmentation experiment consisted of four treatments: (1) soil treated with strain CD 130, (2) soil treated with strain CD 167, (3) soil treated with a combination of the CD 130 and CD 167 strains, and (4) soil treated with sterile saline (0.9%) instead of a bacterial suspension. Bacterial suspensions (30 ml) in sterile saline (0.9%) were poured into the soil to reach a bacterial density of 10^7^ colony forming units (CFUs) g^–1^ d.w. of the soil. Afterwards, soil of each group was gently mixed to obtain an equal distribution of the bacterial suspension or sterile saline in soil. Setup microcosms were prepared in triplicates for each time point and treatment. The experiment was performed for 182 days. The entire soil content was withdrawn from each treatments on days 1, 91 and 182, mixed thoroughly, immediately stored at −80°C and then used for MiSeq analyses.

### DNA Extraction

DNA was extracted from the soils (*n* = 3 for each treatment and duration) using a DNeasy PowerSoil Kit (Qiagen, Hilden, Germany) according to the manufacturer’s instructions. The yield and purity of the DNA was determined using a NanoDrop ND-1000 spectrophotometer (NanoDrop Technologies, Wilmington, DE, United States). The isolated DNA samples were stored at −20°C.

### Illumina MiSeq Sequencing

Amplicon libraries were constructed for the MiSeq platform using bacterial primers 341F (5’-CCTACGGGNGGCWGCAG-3’) and 785R (5’-GACTACHVGGGTATCTAATCC-3’) for the V3-V4 region of the 16S rRNA gene (Klindworth et al., 2013). Each library was prepared using the Q5 Hot Start High-Fidelity DNA Polymerase (NEBNext – New England BioLabs, Ipswich, MA, United States) according to the manufacturer’s instructions. Paired-end (PE, 2 × 250 nt) sequencing was performed on an Illumina MiSeq instrument (MiSeq Reagent kit v2) following the manufacturer’s protocols (Illumina, Inc., San Diego, CA, United States). The automatic primary analysis and the de-multiplexing of the raw reads were performed on MiSeq using the MiSeq Reporter (MSR) v2.4 software (BaseSpace). The sequences were subsequently processed on the CLC Genomics Workbench v8.5.1 (QIAGEN, Aarhus A/S, http://www.clcbio.com). Briefly, the adapter sequences were removed using the Trim Reads tool. Read trimming was performed using the default parameters (trim using quality scores = 0.05 and trim ambiguous nucleotides = 2). Samples with low coverage (low number of reads) were excluded from subsequent analysis. Paired reads were merged and chimeric sequences were removed in the CLC microbial genomics module v1.1 using the default settings. Closed reference operational taxonomic unit (OTU) picking at 97% similarity was also performed. Greengenes v13_5 99% (DeSantis et al., 2006) for 16S rRNA (bacteria and archaea) was used as the reference OTU database. The alpha diversity parameters (OTU, Chao1-bias corrected, Shannon and Simpson indices) were generated using the CLC Microbial Genomics Module v1.1. The sequences generated in this study were deposited in the GenBank SRA database under BioProject accession number PRJNA542795.

### Metagenome Functional Content Prediction

Functional profiles were derived from the 16S sequence data with PICRUSt using the KEGG database as a reference (Langille et al., 2013; Kanehisa et al., 2014; Afgan et al., 2018). Briefly, after normalization, the metagenome was processed and categorized by function to collapse all KEGG orthology (KO)s into KEGG pathways. The results of the functional predictions were subsequently analyzed and visualized in Statistical Analysis of Metagenomic Profiles (STAMP) using the Microbiome Helper (Parks et al., 2014; Comeau et al., 2017). For statistical comparison between predicted pathways, the Welch’s *t*-test and Benjamini-Hochberg *p*-value correction were used. Extended error bar plots generated in STAMP were used to represent these analyses. Chosen KOs were used to determine the contribution of OTUs to specific functions and visualized in MS Excel.

### Statistical Analysis

Statistical analysis was performed using the STATISTICA 12.0 PL software (StatSoft, Tulsa, OK, United States). The effects of bioaugmentation and time on alpha diversity estimators were determined by two-way analysis of variance (ANOVA) followed by a post-hoc comparison of the means using the least significant difference (LSD) test.

The Bray-Curtis dissimilarity coefficients were calculated and principal coordinate analysis (PCoA) was performed to visualize patterns in the OTU-based bacterial community structure. The significance of the variations in the structure of bacterial communities was tested using permutational analysis of variance (PERMANOVA) with Bray-Curtis dissimilarity matrix and 9999 permutations. For the pot experiments, data are represented as the mean ± standard deviation of three replicates.

## Results

### Composition of the Soil Bacterial Communities

NGS analysis of bacterial communities yielded a total of 1,237,637 valid reads for all the analyzed samples. An average of 37,554 (range 23,396–46,064) sequence reads per sample were grouped into OTUs (using 97% minimum similarity) and these ranged from 107 to 722 per community. Details about the sequencing quality and diversity parameters of the studied communities are shown in Table 1. Although most of the sequences were classified into a specific genus, some of the reads could not be classified, possibly due to the limits of the technique. The alpha diversity parameters indicated a significantly (*p* < 0.05) higher bacterial diversity, evenness, and species richness in all the soils on day 1 of the experiment and significantly (*p* < 0.05) lower values of these indices during the experimental period.

**TABLE 1:** High-throughput sequence analysis and alpha-diversity indices of the analyzed treatments.

Day	Treatment	OTUs	Chao1	Shannon	Simpson
1	CD 130	63569^a^	85140^a^	5.160.18^a^	0.920.00^a^
	CD 167	7222^a^	83716^a^	6.310.05^a^	0.950.00^a^
	CD 130 + CD 167	66177^a^	82990^a^	5.700.13^a^	0.970.00^a^
	Control	72295^a^	86693^a^	6.040.35^a^	0.950.01^a^
91	CD 130	30327^b^	32431^b^	3.780.19^b^	0.850.03^b^
	CD 167	35020^b^	38728^b^	3.410.03^b^	0.870.02^b^
	CD 130 + CD 167	31654^b^	34664^b^	4.030.05^b^	0.780.02^b^
	Control	2807^b^	3097^b^	3.730.60^b^	0.830.09^b^
182	CD 130	18013^c^	22312^c^	3.240.53^b^	0.760.10^b^
	CD 167	14850^c^	18255^c^	3.750.04^b^	0.920.01^b^
	CD 130 + CD 167	2454^c^	2687^c^	4.720.18^b^	0.860.01^b^
	Control	1077^*c*^	13212^c^	3.470.08^b^	0.830.01^b^

*± Standard deviation of three independent experiments. Different letters (within each group) indicate significant differences (p < 0.05, LSD test) based on the effects of the time.*

**FIGURE 1:**
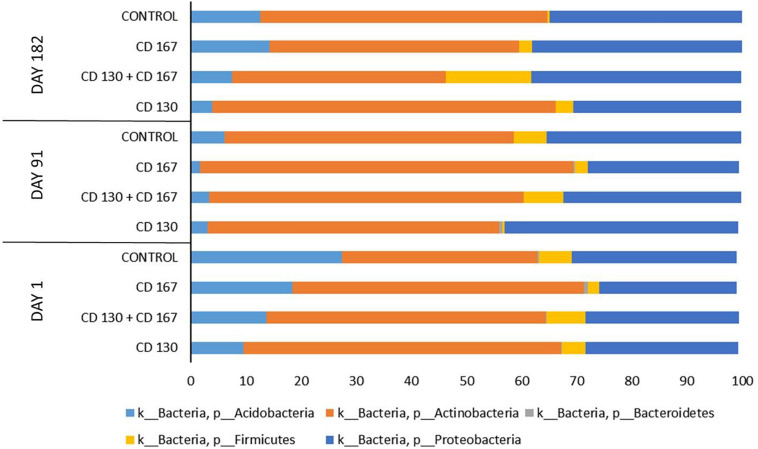
Relative abundance of the main bacterial phyla in the soils from the different treatments during the experimental period.

The taxonomic composition of the bacterial communities in the analyzed soils at the phylum level is shown in Figure 1. In all the samples from all treatment days, *Actinobacteria* and *Proteobacteria* were predominant and accounted for 65–95% of the total bacteria. Members of *Acidobacteria* also had a high relative abundance in the tested soils; however, their percentage varied between the treatments. The sequences classified as belonging to *Firmicutes* and *Bacteroidetes* constituted a small fraction of the total reads in most of the analyzed samples. The only exception was the soil inoculated with the combination (CD 130 + CD 167), in which the percentage of *Firmicutes* on day 182 reached 16%, whereas, in the other samples, it was in the 0–8% range.

**FIGURE 2:**
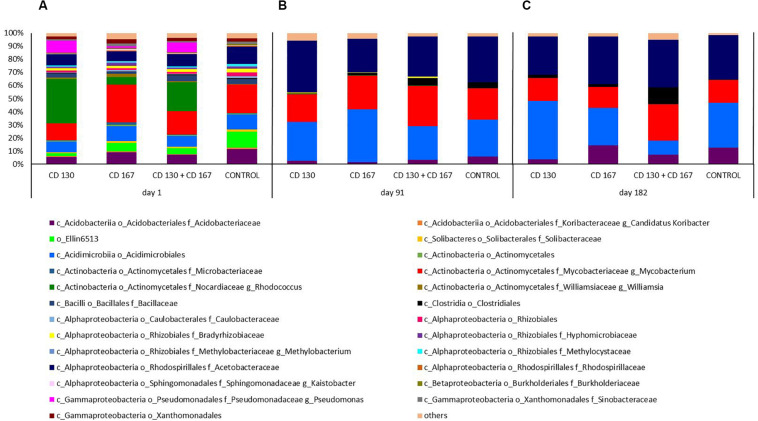
Relative abundance of the bacterial genera in the soil subjected to bioaugmentation on **(A)** day 1, **(B)** day 91, and **(C)** day 182.

A more detailed analysis of the composition of the bacterial communities in the soil at the genus level is shown in Figure 2. On day 1, the predominant genus varied depending on the treatment. In the CD 130 and CD 130 + CD 167 inoculated soils, the genus to which the introduced strains belonged (*Rhodococcus*) was dominant and its relative abundance was 33.89% and 22.28%, respectively. The predominant genus in the CD 167 and control soils was *Mycobacterium*, which accounted for 28.71% and 21.55% of the total reads in these samples, respectively. For comparison, the percentage of the *Mycobacterium* genus in the bacterial community of the CD 130 and CD 167 soils was 12.79% and 17.62%, respectively. On the other hand, the relative abundance of the *Rhodococcus* genus in the soil inoculated with the bacterial combination and in the non-inoculated soil was 5.82% and 0.02%, respectively. In all the bioaugmented soils, the *Pseudomonas* genus was detected (relative abundance 9.39%, 7.28%, and 1.28% in soils CD 130, CD 130 + CD 167, and CD 167, respectively), whereas bacteria from this genus were not detected in the control soil. In all the soils, there was a high relative abundance of reads belonging to the *Acetobacteraceae* and *Acidobacteriaceae* families, the order *Acidimicrobiales*, and the group Ellin6513. Unfortunately, we were unable to assign these reads to specific genera, but interestingly these reads were more abundant in the control soil compared to those in the soils inoculated with bacteria. In all the soils analyzed, bacteria from the *Bacillaceae* and *Bradyrhizobiaceae* families were detected. There was a higher abundance of *Salinibacterium*, *Williamsia*, *Parvibaculum*, *Kaistobacter*, and *Nevskia* in soils treated with the CD 167 strain than in the soils treated with the CD 130, CD 130 + CD 167 strains and the control soil.

Compared to day 1, smaller differences were observed in the composition of the bacterial communities inhabiting the soils on day 91. On this day, the predominance of *Acetobacteraceae* (with relative abundance 39.72%, 30.82%, 25.63%, and 34.82% in soils CD 130, CD 130 + CD 167, CD 167, and the control, respectively) was observed in all the analyzed soils. Furthermore, we observed a high contribution of *Acidimicrobiales* (relative abundance 29.47%, 25.71%, 40.26%, and 27.85% in the CD 130, CD 130 + CD 167, CD 167, and control soils, respectively) and *Actinomycetales*, more specifically sequences belonging to the *Mycobacteriaceae* family and the genus *Mycobacterium* (20.93%, 30.62%, 25.66%, and 23.83% in the CD 130, CD 130 + CD 167, CD 167, and control soils, respectively). Among the *Actinomycetes*, representatives of the family *Nocardiaceae*, the genus *Rhodococcus* were also detected, but their abundance in the CD 130, CD 130 + CD 167, and CD 167 soils was much smaller compared to that on day 1 (0.37%, 0.21%, and 0.23%, respectively); however, it was still higher compared to the control soil. Interestingly, in the soil inoculated with the bacterial combination and in the non-inoculated soil, a higher abundance of *Clostridiales* (6.18% and 5.26%, respectively) with a predominance of the *Clostridium* genus was observed, compared to the CD 130 and CD 167 soils (0.38% and 1.995%, respectively).

Like day 91, a predominance of *Acetobacteracea* (relative abundance 29.23%, 36.42%, 26.37%, and 34.08% in the CD 130, CD 130 + CD 167, CD 167, and control soils, respectively) was also observed on day 182. In all the samples, a high abundance of *Acidimicrobiales* was seen, but the percentage of these reads varied between the treatments. The highest abundance of these orders was observed in the CD 130 soil (44.40%), and the lowest in the soil inoculated with a combination of strains CD 130 and CD 167 (10.57%). In the CD 167 and control soils, the relative abundance of *Acidimicrobiales* was 28.70% and 34.32%, respectively. The soil samples also differed in the abundance of *Acidobacteriaceae* and *Mycobacteriacea*. An increased abundance of *Acidobacteriaceae* was observed in the soil treated with strain CD 167 and the untreated soil compared to that in the others, whereas the abundance of *Mycobacteriaceae* was higher only in the soil inoculated with the combination. The abundance of *Clostridiales* (in particular, the genera *Clostridium, Thermoanaerobacterium*, and *Desulfosporosinus*) was higher in the soil inoculated with the bacterial combination (14.68%), whereas in the CD 130, CD 167, and control soils, it only reached 2.91%, 2.16%, and 0.41%, respectively.

**FIGURE 3:**
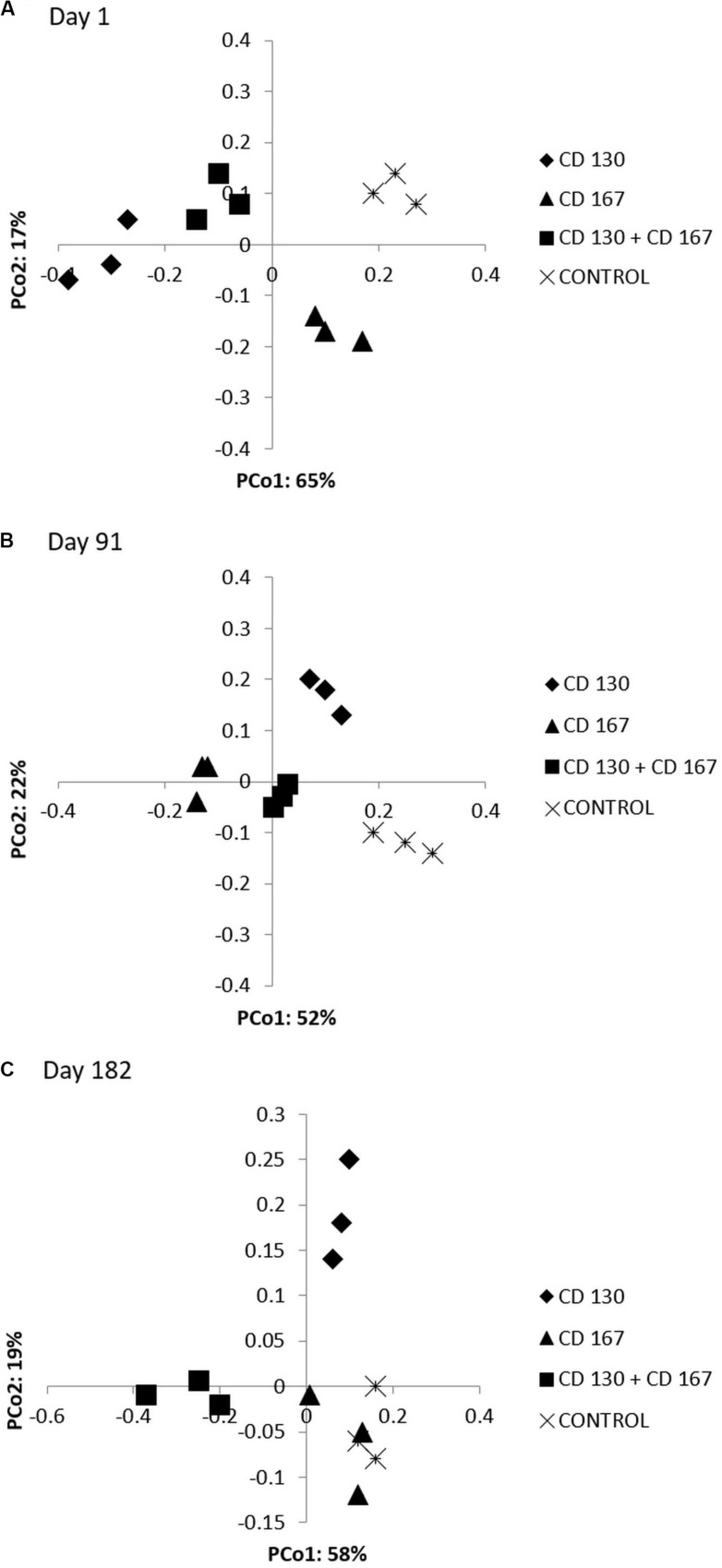
Principle coordination analysis (PCoA) plots based on the Bray-Curtis similarities of the OTU-based bacterial community analysis of the soils subjected to bioaugmentation on **(A)** day 1, **(B)** day 91, and **(C)** day 182.

To visualize the differences in the composition of bacterial communities between various soils during the experiment, a PCoA based on the Bray-Curtis dissimilarity matrix was performed. The PCoA ordination showed that at the beginning of the experiment, the composition of the bacterial communities in soils bioaugmented with strain CD 130 and the combination differed from the control and CD 167 soils (Figure 3A). On day 91, differences in the biodiversity of the bacterial communities were observed between soils subjected to bioaugmentation and the control soils (Figure 3B). Nevertheless, at the end of the experiment, the composition of the bacterial communities did not vary significantly between the soils treated with strains CD 130, CD 167, and the control soils. Differences in the structure of the bacterial communities were still observed between the control and CD 130 + CD 167 soils (Figure 3C). PERMANOVA analysis confirmed the significance of clustering (day 1: *p* = 0.0002, day 91: *p* = 0.0427, day 182: *p* = 0.0003); however, with three replicates for each sample, the clustering was not significant with pair-wise comparisons of the types.

### PICRUSt Analysis

To perform a comprehensive analysis of the functional composition of the metagenome of each soil microbial community based on its 16S rRNA sequences, PICRUSt method was used. This allowed us to establish a connection between the phylogenetic structure and the metabolic profiles of the soil microbiomes during the bioaugmentation experiment.

The PICRUSt tool enabled the prediction of potential KEGG pathways and then STAMP analysis was used to quantify significant differences in the abundance of the predicted pathways between different treatments. One day after treatment with CD 130, CD 167 or their combination, a significant effect on 83%, 55%, and 78%, respectively, of the predicted functional pathways was observed (Supplementary Figures S1–S3). A significantly higher abundance of xenobiotic biodegradation and metabolism, lipid metabolism, metabolism of terpenoids and polyketides, amino acid metabolism, and metabolism of special amino acids was seen in all bioaugmented soils than in the control soil. On day 91, there were no significant differences in the predicted functions between treated and untreated soils. At the end of the experiment, significant differences in the abundance of KEGG pathways were detected between the control, CD 130, and CD 130 + CD 167 soils. In case of the CD 130 soil, abundance of only two functions, protein translation and metabolism of terpenoids and polyketides, was changed (Supplementary Figre S4). However, 62% of the predicted functional pathways were significantly changed at the end of the experiment in soil inoculated with the CD 130 + CD 167 strain compared to those in the control (Supplementary Figure S5).

**FIGURE 4:**
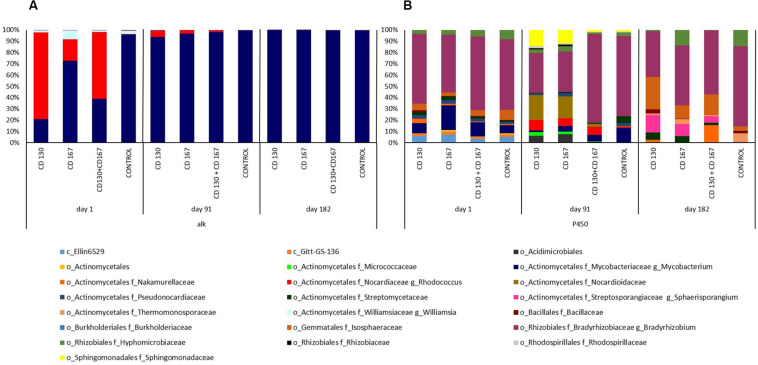
Predicted metagenome contributions to **(A)** *alkH* and **(B)** *CYP153* genes in soils from different treatments.

To better understand the changes in the composition of bacteria involved in the degradation of hydrocarbons, the association between bacterial taxa and following genes: *alkH* (K00496), *CYP153* (K14338), *C120* (K03381), and *C230* (K00446) was analyzed. At the beginning of the experiment, the *alkH* gene was contributed by bacteria from the *Rhodococcus* and *Mycobacterium* genera in bioremediated soils, whereas in control soil the *Mycobacterium* genus was the predominant contributor. On days 91 and 182, abundance of this gene was contributed mainly by the *Mycobacterium* genus in all soils (Figure 4A). The taxonomic composition for NADPH-cytochrome P450 reductase (K14338), catechol 1,2-dioxygenase (K03381), and catechol 2,3-dioxygenase (K00446) had different patterns in soils from different treatments during the entire experimental period. For NADPH-cytochrome reductase, the most obvious changes in bacterial contributions between soils from different treatments were observed on days 91 and 182. On day 91, a higher abundance of *Bradyrhizobiaceae* was observed in the control and CD 130 + CD 167 soils (70.77 and 78.63%, respectively) compared to that in the CD 130 and CD 167 soils (35.11 and 35.87%, respectively). However, a smaller abundance of *Sphingomonadaceae* was observed in the control and CD 130 + CD 167 soils (1.72 and 2.05%, respectively) compared to that in the CD 130 and CD 167 soils (14.38 and 11.90%, respectively). Additionally, on this day, a higher abundance of *Nocardiaceae* (especially *Rhodococcus*) and a lower abundance of *Streptomycetaceae* were observed in all bioremediated soils. On day 182, the taxonomic contribution for the *CYP153* gene had different patterns in soils from different treatments (Figure 4B).

**FIGURE 5:**
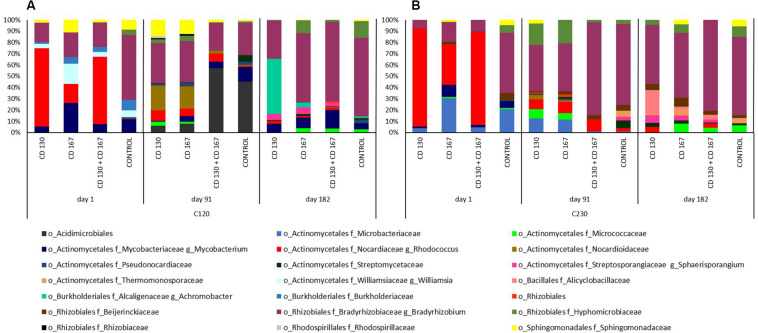
Predicted metagenome contributions to **(A)** *C120* and **(B)** *C230* genes in soils from different treatments.

Differences in the bacterial contributions of the *C120* gene, reported on day one, were mainly due to the higher abundance of *Nocardiaceae* (especially *Rhodococcus)* in soils CD 130, CD 167, and CD 130 + CD 167 (69.32, 17.06, and 59.89%, respectively) and lower abundance of *Bradyrhizobiaceae* (17.25, 21.95, and 21.35%), compared to the control soil, where the abundance of *Nocardiaceae* was 0.28%, and abundance of *Bradyrhizobiaceae* was 57.52%. On day 91, similarities in the patterns of bacterial taxonomic composition for the *C120* gene were observed between soils CD 130 and CD 167, and between soil treated with the bacterial combination and the control. At the end of the experiment, the soil CD 130 had a different taxonomic profile, compared to that in soils CD 167, CD 130 + CD 167, and the control. In the soil CD 130, *Mycobacteriacea, Streptosporangiaceae, Alcaligenaceae*, and *Bradyrhizobiacea* were the main contributors of the *C120* gene, whereas in the other soils, the biodiversity of bacteria contributing to the abundance of this gene was higher (Figure 5A).

The taxonomic contribution for the *C230* gene on day one had a similar pattern in soils CD 130 and CD 130 + CD 167. In these soils, the *C230* gene was mainly contributed by *Nocardiaceae*, *Bradyrhizobiaceae*, and *Microbacteriacea* (abundance 87.04%, 7.61%, and 4.03%, respectively, in soil CD 130 and 82.51%, 10.40%, and 4.75%, respectively, in soil CD 167). On day one, the abundance of the *C230* gene in soil CD 167 was mainly contributed by *Nocardiaceae* (36.30%), *Microbacteriaceae* (30.64%), *Bradyrhizobiaceae* (17.80%), and *Mycobacteriaceae* (10.77%), as opposed to *Bradyrhizobiaceae* (53.27%), *Microbacteriacea* (20.98%), *Mycobacteriaceae* (6.37%), and *Beijerinckiaceae* (6.09%) in the control soil. On day 91, like the *C120* gene, similarities in the taxonomic patterns for the *C230* gene were observed between soils CD 130 and CD 167, and between the soil treated with the bacterial combination and the control. On day 182, the main contributor of the *C230* gene in all soils was *Bradyrhizobiaceae;* however, the abundance of the other taxonomic groups differed between treatments (Figure 5B).

It should be emphasized that the PICRUSt analysis showed that among the genes involved in hydrocarbon degradation that were tested, the abundance of the *alkH* gene was 1–2 orders of magnitude higher than *CYP153, C120, and C230*.

## Discussion

In the current investigation, changes in the taxonomic and functional composition of the bacterial communities in an aged petroleum hydrocarbon-contaminated soil subjected to bioaugmentation with the hydrocarbon-degrading strains *R. erythropolis* CD 130 and CD 167 or their combination were studied. Such analyses are crucial since we still do not know whether the loss of hydrocarbons in bioaugmented soils occurs due to the activity of introduced bacteria or from the increased degradative activity of indigenous microorganisms. The key question is whether inoculants change the diversity and/or activity of the indigenous bacterial communities resulting in higher hydrocarbon removal.

The soil used in the bioaugmentation experiment was characterized by a very low pH (pH = 4.02) and a very high initial TPH concentration (11980.75 mg kg ^– 1^ d.w. of soil). As a result of applied bioaugmentation causing the changes in the structure of autochthonous bacteria, a 38.40%, 29.8%, and 29.72% hydrocarbon removal was observed on day 182 in soils CD 167, CD 130 and CD 130 + CD 167, respectively, while in the untreated soil, the decrease in TPH was only 14.77%. The observed alterations were related to the increased expression levels of the genes responsible for degradation of hydrocarbons (*CYP153*, *alkH*, and *C230*) in the treated soils. Moreover, changes in the patterns of phospholipid fatty acids characteristic for gram-positive and gram-negative bacteria were also observed. The bioaugmentation experiment, as well as determination of gene expression and phospholipid fatty acid analysis, were performed and described in Pacwa-Płociniczak et al., 2019. Those results suggested that the observed changes accelerated the removal of hydrocarbons from this soil; however, more detailed studies on specific bacterial dynamics were needed. Thus, in this study, a comprehensive analysis was performed on soils subjected to bioaugmentation, including analysis of taxonomic composition and prediction of the functional composition of the metagenome of the bacterial communities from its 16S rRNA sequences.

These results indicated that in the laboratory microcosms values of alpha-diversity indices declined. The decrease in OTUs, Chao1, Shannon and Simpson values was found in both inoculated and control soils, which proved that the separation of the soil into the microcosms altered its physicochemical characteristics and changed the biodiversity of soil bacterial communities. Similarly, a gradually decreasing in the values of alpha-diversity indices with time in the soil microcosms was observed by other authors (Sun et al., 2014; Nicolitch et al., 2019).

Metagenomic analysis showed that *Acidobacteria, Actinobacteria, Proteobacteria*, and *Firmicutes* were the predominant phyla present in the aged petroleum-hydrocarbon contaminated control soil on day 1 of the experiment. Similar to our results, members of above-mentioned phyla, especially representatives of *Acidobacteriaceae* and the group Ellin6513 within *Acidobacteriacea, Acidimicrobiales*, and *Actinomycetales* (particularly genus *Mycobacterium*) within *Actinobacteriaceae*, were highly enriched in acidic soil samples (pH 4.26–4.4) contaminated with polycyclic aromatic hydrocarbons (PAH) (Wu Y. et al., 2017). Their studies revealed that pH had a profound impact on the composition of the bacterial community in PAH-polluted soil (Wu Y. et al., 2017).

In contrast to our experiment, in which the *Alphaproteobacteria* were predominant, Koshlaf et al. (2016) and Czarny et al. (2020), reported that the *Gammaproteobacteria* dominated petroleum-hydrocarbon contaminated soil. Members of this class, primarily *Pseudomonadaceae*, are known to be effective degraders of petroleum hydrocarbons (Saikia et al., 2012), and they are often found in soils contaminated with PAHs (Wu M. et al., 2017). In our investigation, *Gammaproteobacteria* were present in all treated soils on day 1, however, in the control soil, they were represented only by the members of the *Cyanobacteria* and *Xanthomonadaceae* families. Similarly, *Xanthomonadaceae* were the predominant representative of *Gammaproteobacteria* in acidic PAH-contaminated soils (Wu Y. et al., 2017). In our experiment, members of the *Pseudomonadaceae* were detected in all bioaugmented soils; however, their abundance differed between treatments. Additionally, it seems that bacteria from this family do not degrade hydrocarbons because they did not contribute to the abundance of the *alkH, CYP153, C120*, and *C230* genes. On day 1, in the bioaugmented soils, a higher abundance of *Rhodococcus* was observed, although this was because of the introduction of bacteria from this genus. Interestingly, in the control soil, the abundance of *Rhodococcus* was low, even though bacteria from this genus dominated the culturable hydrocarbon-degrading bacteria isolated from the same soil (Pacwa-Płociniczak et al., 2016b).

Furmanczyk et al. (2017) found that all the sodium dodecyl sulfate-degrading bacterial strains isolated from peaty soil were classified into the genus *Pseudomonas*, whereas a metagenomic analysis showed that only 0.58% of the sequences were assigned to the *Pseudomonas* genus. We hypothesized that bacteria from the *Rhodococcus* genus, dominated the fraction of hydrocarbon-degrading strains able to grow under laboratory conditions; however, they were not the most effective hydrocarbon-degraders in the community. Confirmation of this hypothesis was found by assessing the contribution of the bacterial taxa to the abundance of the *alkH* gene. High contribution (in the range of 18.99–76.82%) of the *Rhodococcus* genus to the abundance of this gene was observed only on day one and in the soils subjected to bioaugmentation. On day 91, this contribution was in the range of 1.02–3.36%, while on day 182, members of the *Rhodococcus* genus were not detected among bacterial genera contributing to the abundance of the *alkH* gene. Similarly, rifampicin-resistant mutants of the introduced *Rhodococcus* strains were detected in soils for only 42 days (Pacwa-Płociniczak et al., 2019) confirming that the introduced strains did not survive in the soil. Because hydrocarbon removal in the bioaugmented soils after day 91 still was higher than in the control, we hypothesized that the autochthonous bacteria were responsible for the increased hydrocarbon removal. This was also confirmed by the results of the taxonomic contribution of *alkH* in the soils tested. We found that the genus *Mycobacterium*, which was the main contributor of the *alkH* gene in the control soil during the entire experimental period, also dominated all bioaugmented soils from day 91 to the end of the experiment. Li et al. (2018) observed that the introduction of *Acinetobacter tandoii* LJ-5 into PAH-contaminated wastewater resulted in a significant increase in the biodegradation of phenanthrene (PHE), but LJ-5 did not participate directly in PHE degradation. The improved biodegradation of PHE was attributed to the remarkably altered diversity of PHE degraders. Similarly, in our study, hydrocarbon degradation was the result of changes observed in the composition of the indigenous bacterial communities, especially those contributing to the abundance of *CYP153, C120*, and *C230* genes, in soils inoculated with the CD 130 and/or CD 167 strains. Additionally, PICRUSt analysis showed that soil inoculation significantly increased the potential of microbial communities to metabolize xenobiotics, lipids, terpenoids, polyketides, and amino acids on day one of the experiment. Differences in the abundance of KEGG pathways were also detected on day 182 between the control, CD 130, and CD 130 + CD 167 soils. These differences were also connected with the bacterial metabolic functions in the soil and could have positive impact on the hydrocarbon removal in the bioaugmented soils. Moreover, the dead biomass of inoculants could be a source of nutrients for the autochthonous bacteria and thus provide a biostimulation effect. Similarly, Koshlaf et al. (2016) observed shifts in bacterial communities of bioaugmented diesel-contaminated soil compared with the original soil. Siles and Margesin (2018) reported that the changes in the structure of the bacterial and fungal communities correlated with the changes in the TPH content; however, the changing abundance of bacteria and fungi did not play a key role on the effectiveness of soil bioremediation. Guo et al. (2017) also observed that a pyrene-degrading bacterial consortium introduced into pyrene- and benzo[α]pyrene-contaminated soil could not compete for resources with the indigenous microorganisms and nearly disappeared after ten weeks. While non-indigenous bacterial strains were used in their experiment, bacterial strains that had previously been isolated from tested soil were used in our experiment but still were unable to survive.

## Conclusion

The presented analyses of the taxonomic and functional composition of bacteria in soil contaminated with petroleum-hydrocarbons subjected to bioaugmentation provides a better understanding of the dynamics of autochthonous bacterial communities. The results revealed that a high proportion of the *R. erythropolis* CD 130 and CD 167 strains were present in the inoculated soils only at the beginning of the experiment, which indicates that they did not survive in the bioaugmented soil. This finding suggests that hydrocarbon loss in the analyzed soil resulted from the activity of autochthonous microorganisms. Nevertheless, the observed hydrocarbon degradation ultimately occurred due to changes in the composition and functional activity of the indigenous bacterial community induced by the CD 130 and/or CD 167 strains. The analysis confirmed our hypothesis that the application of CD 130 and CD 167 strains singly or in combination cause temporary changes in the genetic and functional structures of soil autochthonous bacterial communities.

## Data Availability Statement

The datasets presented in this study can be found in online repositories. The names of the repository/repositories and accession number(s) can be found below: https://www.ncbi.nlm.nih.gov/genbank/, PRJNA542795.

## Author Contributions

MP-P designed and performed bioaugmentation experiment, performed the DNA isolation from soil, conducted the statistical analyses, analyzed and interpreted all results, and wrote the manuscript. PB performed bioaugmentation experiment. KB conducted NGS and PICRUSt analyses. ZP-S was involved in the writing of the manuscript. All authors contributed to the article and approved the submitted version.

## Conflict of Interest

The authors declare that the research was conducted in the absence of any commercial or financial relationships that could be construed as a potential conflict of interest.

## Supplementary Material

FIGURE S1 Extended error bar plots showing the mean proportion (%) of significantly different predicted functional categories at level 2 between soil CD 130 and control on day one.

FIGURE S2 Extended error bar plots showing the mean proportion (%) of significantly different predicted functional categories at level 2 between soil CD 167 and control on day one.

FIGURE S3 Extended error bar plots showing the mean proportion (%) of significantly different predicted functional categories at level 2 between soil CD 130 + CD 167 and control on day one.

FIGURE S4 Extended error bar plots showing the mean proportion (%) of significantly different predicted functional categories at level 2 between soil CD 130 and control on day 181.

FIGURE S5 Extended error bar plots showing the mean proportion (%) of significantly different predicted functional categories at level 2 between soil CD 130 + CD 167 and control on day 181.
