# Supplementary figures and images for: Metagenomic Functional Profiling Reveals Differences in Bacterial Composition and Function During Bioaugmentation of Aged Petroleum-Contaminated Soil
Source: Front Microbiol. 2020 Aug 31;11:2106. doi: 10.3389/fmicb.2020.02106 (PMC7487420; doi:10.3389/fmicb.2020.02106)

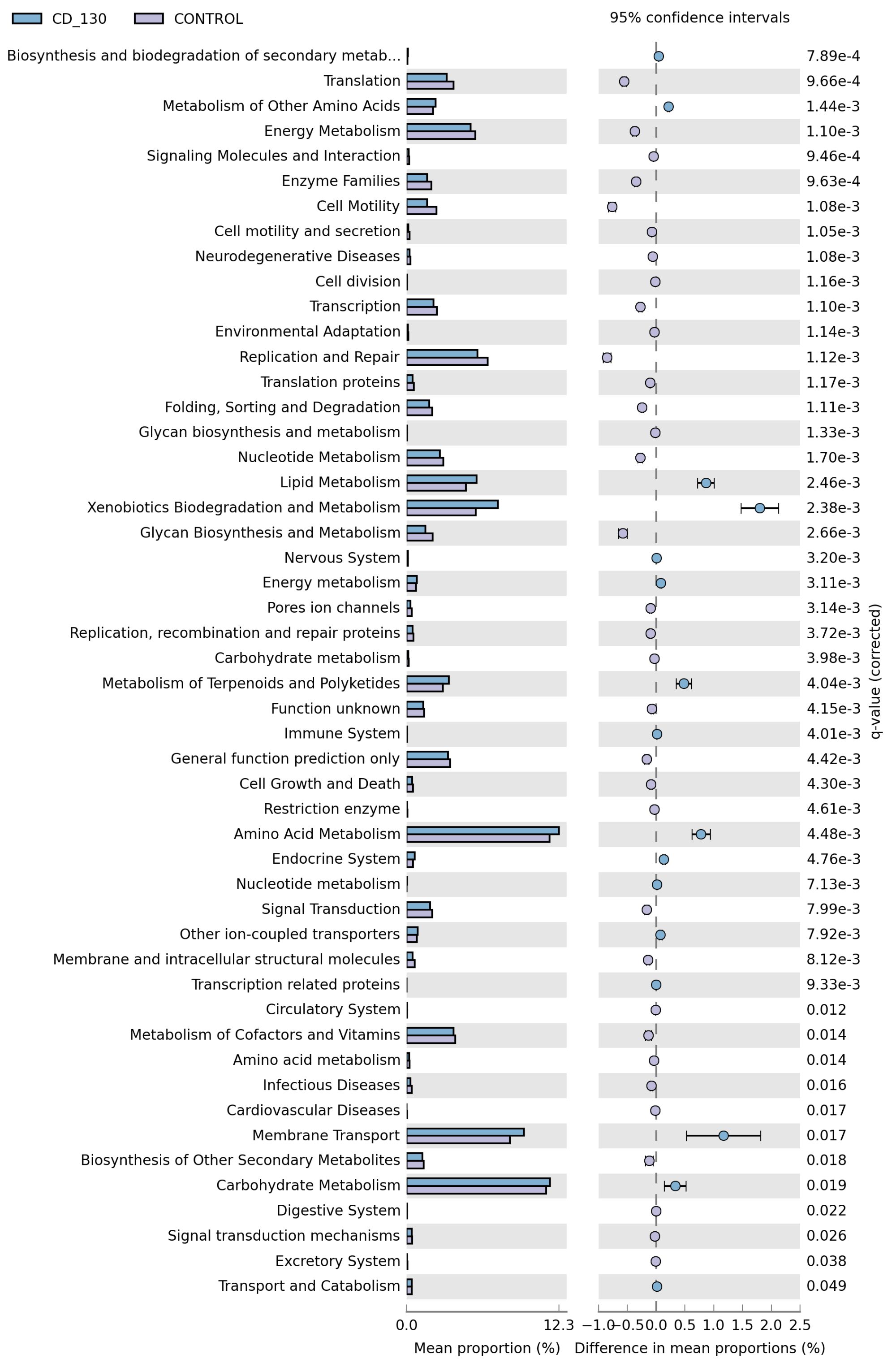

Supplement: FIGURE S1 — Extended error bar plots showing the mean proportion (%) of significantly different predicted functional categories at level 2 between soil CD 130 and control on day one. [file Image_1.JPEG]

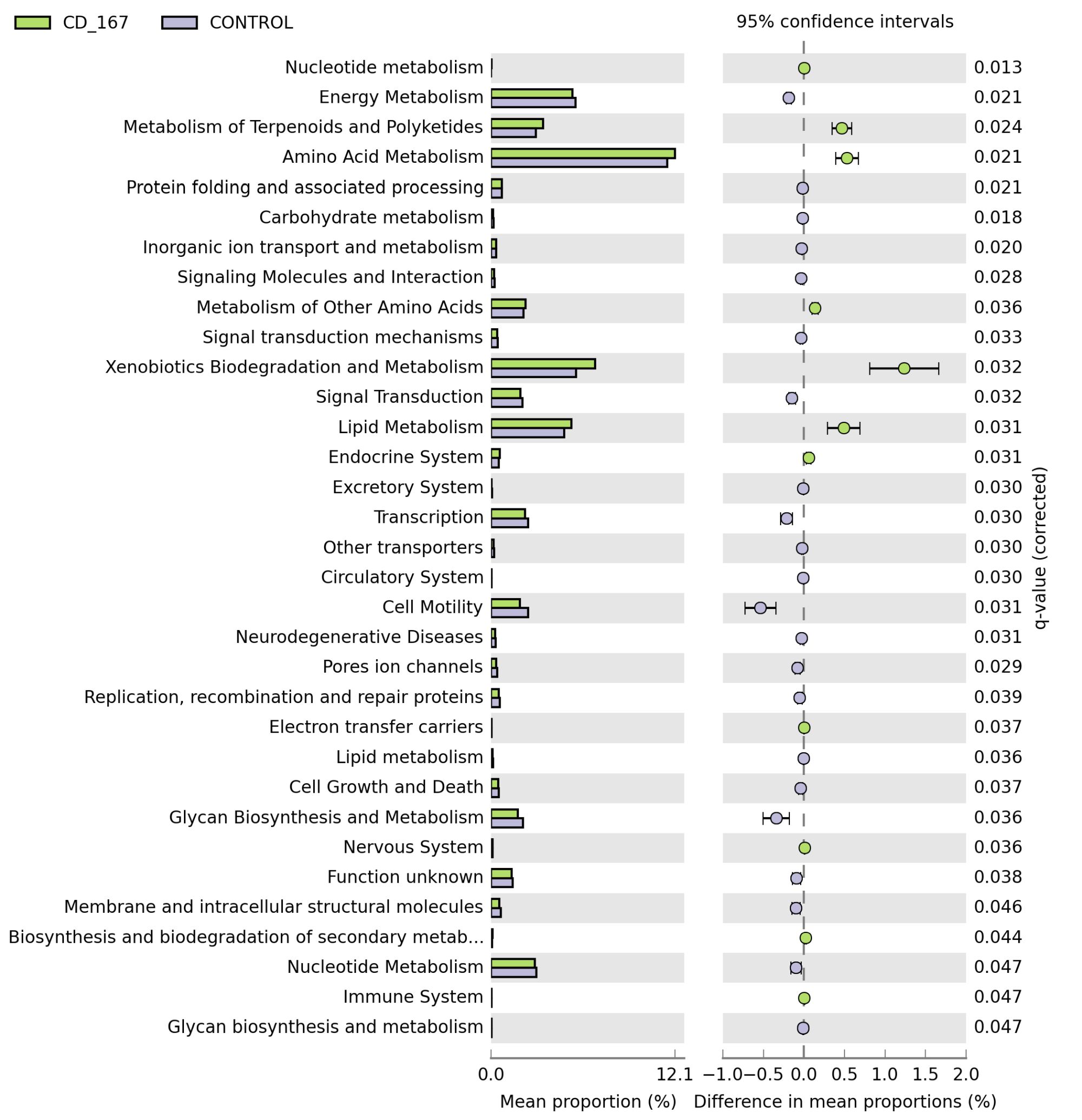

Supplement: FIGURE S2 — Extended error bar plots showing the mean proportion (%) of significantly different predicted functional categories at level 2 between soil CD 167 and control on day one. [file Image_2.JPEG]

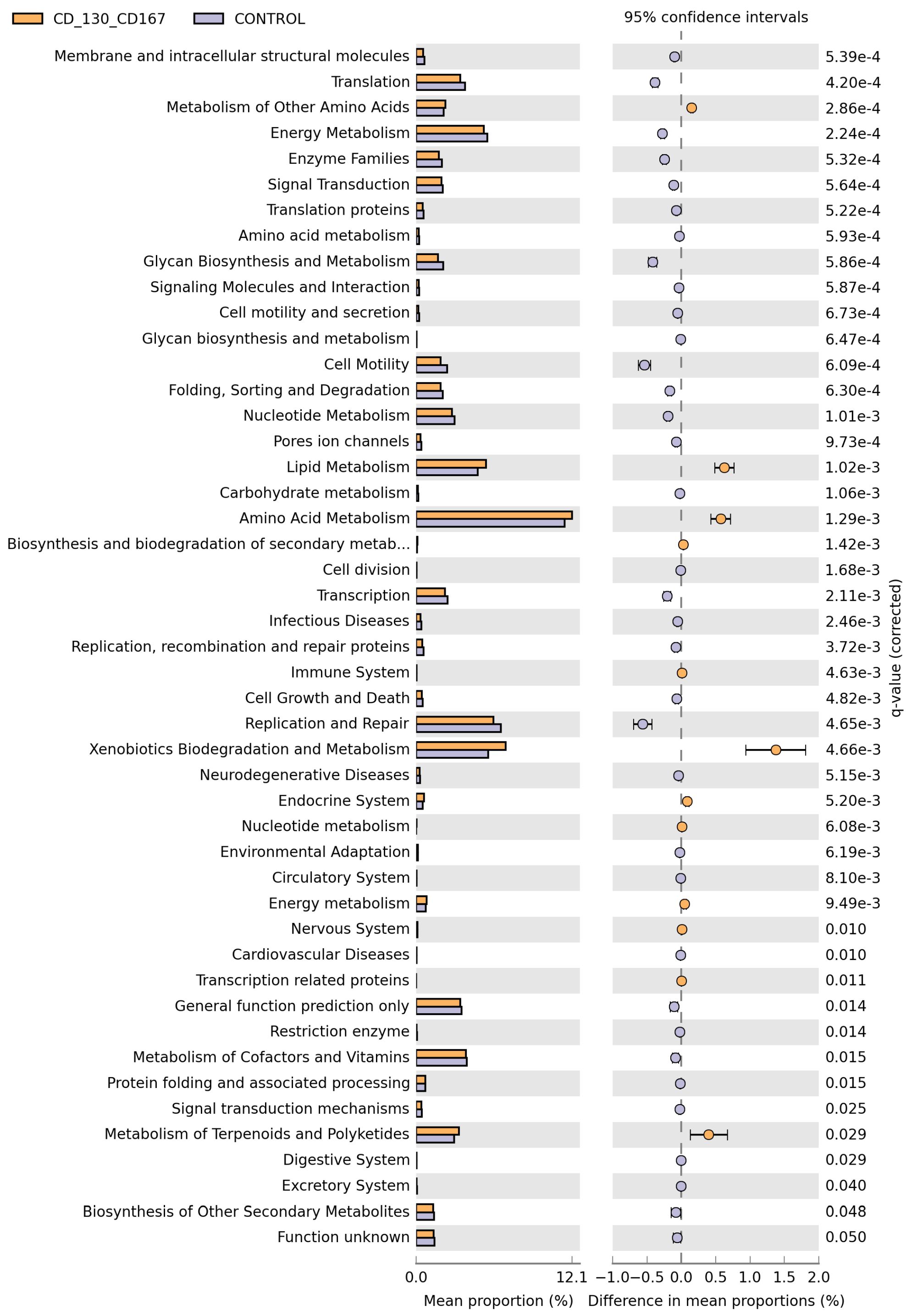

Supplement: FIGURE S3 — Extended error bar plots showing the mean proportion (%) of significantly different predicted functional categories at level 2 between soil CD 130 + CD 167 and control on day one. [file Image_3.JPEG]

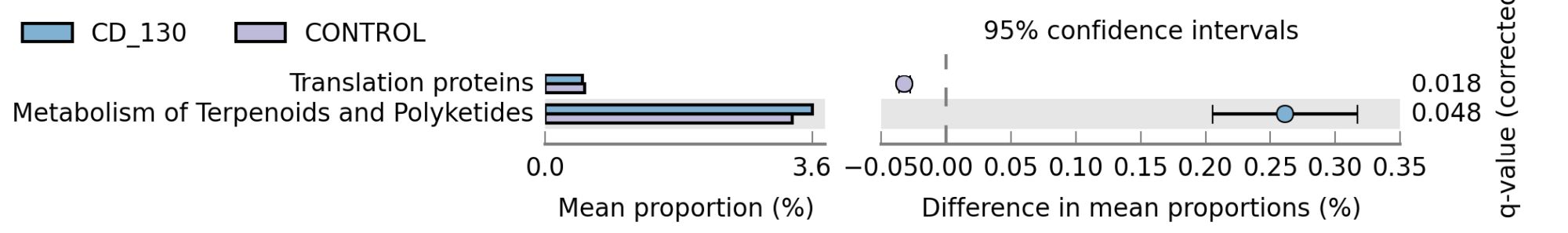

Supplement: FIGURE S4 — Extended error bar plots showing the mean proportion (%) of significantly different predicted functional categories at level 2 between soil CD 130 and control on day 181. [file Image_4.JPEG]

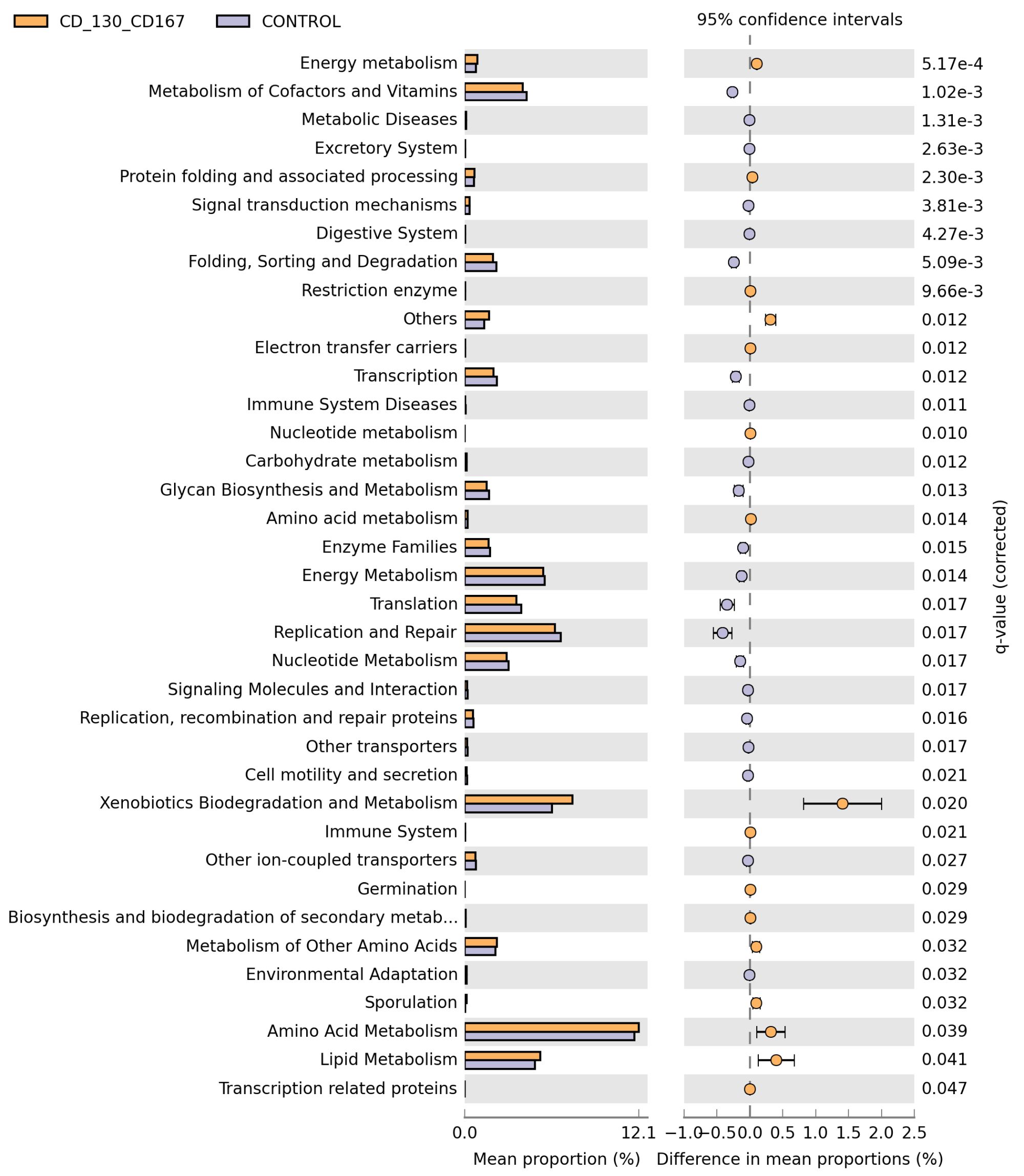

Supplement: FIGURE S5 — Extended error bar plots showing the mean proportion (%) of significantly different predicted functional categories at level 2 between soil CD 130 + CD 167 and control on day 181. [file Image_5.JPEG]
